# The Association Between High Levels of Aggression and Insomnia in Chinese Adolescents: A Longitudinal Latent Profile Analysis

**DOI:** 10.1155/da/3713624

**Published:** 2025-05-14

**Authors:** Sihong Li, Hui Chen, Xianliang Chen, Huajia Tang, Yanyue Ye, Jiansong Zhou

**Affiliations:** Department of Psychiatry, National Clinical Research Center for Mental Disorders, and National Center for Mental Disorders, The Second Xiangya Hospital of Central South University, Changsha, Hunan, China

**Keywords:** adolescents, aggression, cohort study, insomnia, latent profile analysis

## Abstract

**Background:** Aggression has been reported to be associated with insomnia in adolescents. However, the impact of aggression and different levels of aggression on insomnia needs further exploration. This study aimed to explore the association of aggression, as well as different profiles of aggression, with insomnia in Chinese adolescents.

**Method:** This was a prospective cohort study with an 8-month follow-up period. The Short-Form Buss–Perry Aggression Questionnaire (BPAQ-SF) was used to assess the aggression; the Insomnia Severity Index (ISI) was used to assess the symptoms of insomnia. Latent profile analysis (LPA) was conducted to identify profiles of aggression. The association between different profiles of aggression and insomnia was assessed using logistic regression analysis. We also used the restricted cubic spline model to investigate the pattern of the association.

**Results:** A total of 1124 students completed the questionnaire. The aggression was classified into three profiles: low aggression (*n* = 931, 82.8%), moderate aggression (*n* = 153, 13.6%), and high aggression (*n* = 40, 3.6%). A follow-up survey after 8 months found 228 (20.3%) new cases of insomnia. Moreover, high aggression was associated with a significantly increased risk of insomnia after adjustment for age, sex, ethnicity, anxiety state, and depressive state (odds ratio [OR]: 9.98, 95%CI: 4.94–20.15). The relationship between aggression and the risk of insomnia was linear in the restricted cubic spline regression analysis.

**Conclusion:** High levels of aggression were significantly associated with insomnia among Chinese adolescents. Therefore, targeted interventions aimed at addressing insomnia among adolescents with high levels of aggression are needed to improve their mental well-being.

## 1. Introduction

Mental health issues among adolescents have become a global concern [1], with insomnia being recognized as a particularly common and impactful problem [2]. Insomnia, defined as difficulty in initiating or sustaining sleep, is linked to a range of adverse outcomes, including mood disorders, cognitive impairment, physical health problems, and reduced life expectancy [2]. The prevalence of insomnia among adolescents varies significantly, ranging from 7% to 40%, depending on the diagnostic criteria, population, and research methods used [3–5]. In China, research indicates that ~26.9% of school-aged adolescents experience insomnia symptoms, with the prevalence increasing to 31.8% among senior high school students [6]. The high prevalence and severe consequences of insomnia necessitate the identification of factors that increase the risk of developing this condition.

Aggression is another pressing issue during adolescence, often manifesting as a response to interpersonal conflicts and environmental stressors [7]. Adolescence is a period marked by significant physical, emotional, and social development, making individuals particularly prone to exhibiting aggressive behaviors [8]. During this critical stage, the adolescent brain undergoes gradual maturation, and dysfunction in brain regions involved in impulse control, emotional regulation, and sensation-seeking may contribute to increased aggression [9, 10]. Additionally, common challenges faced during adolescence, such as peer conflicts and strained parent–child relationships, can exacerbate emotional difficulties, further elevating the likelihood of aggression [11]. Studies have shown that around 10% of adolescents are diagnosed with disruptive behavior disorders involving aggressive tendencies, such as conduct disorder and oppositional defiant disorder [12]. Specifically, among the subtypes of aggression, verbal and physical aggression are two major dimensions of external behaviors that are easily observable. In the United States, 33% of adolescents report engaging in physical aggression, while 68% experience verbal harassment [13]. Similarly, among Chinese adolescents, the prevalence of aggressive behavior ranges between 15.7% and 24.3% [14, 15]. These findings highlight the widespread nature of both verbal and physical aggression in adolescents. Given that verbal and physical aggression often co-occur, it is crucial to assess them in combination to fully understand their psychological impact.

Aggression in youth has demonstrated continuous trajectory into adulthood and has been a considerable factor associated with psychological issues [16]. Specifically, it has been proposed that sleep is a relevant factor in aggression. Prior meta-analyses and systematic reviews indicated sleep disturbance is significantly associated with increased both verbal and physical aggression [17, 18]. Clinical studies involving children and adolescents have demonstrated a significant correlation between sleep deprivation and increased aggression, alongside difficulties in self-regulation [19, 20]. Furthermore, addressing sleep-related issues has been correlated with notable improvements in behavioral problems [21]. Moreover, sleep quality—such as insomnia—appears to be more closely associated with aggression than sleep quantity [22]. A significant association was found between poor subjective sleep quality and increased aggression in adolescents from the UK Millennium Cohort [23]. This is because poor sleep quality leads to greater impairments in the regulation of cognitive processes, emotions, and subsequent behaviors [18]. Furthermore, according to the hyperarousal theory, heightened arousal serves as a primary mechanism underlying the development of insomnia [24]. Engaging in aggressive behavior may further exacerbate physiological arousal and stress levels, thereby impairing sleep quality [25]. Although many studies have investigated the relationship between sleep disturbances and aggression, they are limited in their ability to establish causal relationships, which are essential for developing targeted interventions. While previous longitudinal studies have explored how sleep problems contribute to aggression, few have examined how aggression may increase the risk of developing insomnia. Addressing this gap is crucial for advancing our understanding of the complex, bidirectional relationship between aggression and sleep, potentially opening new avenues for interventions targeting both aggression and sleep disturbances. Moreover, limited attention has been given to the varying severity and co-occurrence of verbal and physical aggression, which may have differential effects on sleep and aggression trajectories.

To address these research gaps, latent profile analysis (LPA) offers a robust analytical approach by identifying distinct co-occurrence patterns and grouping individuals into profiles based on external variables [26]. For instance, one study combined self-harm and aggression in LPA and identified four profiles, among which 80.4% are low symptoms, 14.2% are moderate aggression, and 3.0% and 2.4% are high aggression and high self-harm, respectively [27]. Another study examining aggression trajectories in middle childhood identified four classes—low aggression, high proactive–reactive aggression, declining aggression, and predominantly reactive aggression—and explored their relationships with outcomes in early adolescence [28]. These findings illustrate the importance of using LPA to uncover nuanced patterns beyond what traditional assessment scales can reveal.

Given the prevalence of aggression and insomnia among adolescents, along with their close association, addressing aggression may represent a previously overlooked intervention target for reducing the risk of insomnia. To fill these gaps in the literature, the present study adopts a prospective cohort design and applies LPA to investigate the association between aggression and the risk of insomnia among Chinese adolescents. This study also investigates how different severity levels and patterns of aggression (e.g., verbal and physical) influence the development of insomnia.

## 2. Methods

### 2.1. Participants

Using a cluster sampling method, this study recruited 1793 first-year students from a vocational school in Changsha (Hunan Province, China) in October 2022 using the following inclusion criteria: (a) school students aged between 18 and 25 years and (b) being able to understand and complete the questionnaire. Students who were absent due to sickness or other reasons during the research period and those who were not able to complete the questionnaire due to severe physical and mental illness were excluded. Exclusion criteria for the follow-up part included insomnia that occurred at baseline. Professionally trained psychiatric personnel were involved in the data collection process to assist students with accessing and completing the questionnaire. They were trained to clarify any instructions or questions the students had, ensuring full understanding of the content. However, they did not participate in interpreting the answers or influencing how the students responded. The survey was administered during scheduled psychology classes at the school, creating a structured environment for the data collection process. The information and responses from all participants were gathered using the Chinese online platform “Sojump” (http://www.sojump.com/). The survey was accessed by the participants through their mobile devices, and they gave electronic informed consent on the initial page of the survey.

This research formed a part of the China Depression Cohort Study (CDCS) and received approval from the Ethics Committee at the Second Xiangya Hospital, affiliated with Central South University. During the baseline assessment in October 2022, 1793 participants were enrolled and completed the survey regarding sociodemographic characteristics, depression, anxiety, aggression, and insomnia. The follow-up survey was conducted 8 months later in June 2023. Students with insomnia at baseline were excluded from the follow-up period, and 1660 of the students agreed to complete the follow-up survey on insomnia. Among them, 536 participants were excluded from the follow-up study due to insomnia found at baseline; thus, a total of 1124 students were included in the follow-up analysis.

### 2.2. Measurement

#### 2.2.1. Sociodemographic Variables

Sociodemographic characteristics, including age, sex, and ethnicity, were collected from participants using a self-designed questionnaire.

#### 2.2.2. Depression

The Patient Health Questionnaire (PHQ-9) was utilized to evaluate the depressive states, serving as a self-reported instrument to measure depression experienced over the preceding 2 weeks [29]. The nine items rating between 0 (not at all) and 3 (almost daily) produce a cumulative score that spans from 0 to 27 [29]. The cutoff score was 10, indicating that a participant with a total score of 10 or above had moderate to severe depression [29, 30]. The Chinese version of PHQ-9 had a Cronbach's alpha of 0.86, suggesting its good reliability and validity [30]. The PHQ-9 showed a Cronbach's alpha of 0.85 in the current study.

#### 2.2.3. Anxiety

The anxiety states of participants in past 2 weeks were evaluated using the 7-item Generalized Anxiety Disorder Scale (GAD-7). This scale is a useful tool with good validity and reliability in identifying general anxiety disorders. Each item is assessed on a scale from 0 (not at all) to 3 (almost every day), with a cumulative score that varies from 0 to 21 [31]; the total score of 10 or above indicates the presence of anxiety [31]. The GAD-7 showed a Cronbach's alpha of 0.88 in the current study.

#### 2.2.4. Insomnia

The Insomnia Severity Index (ISI) is a self-report questionnaire consisting of seven items, utilized for evaluating the severity of insomnia experienced over the past month. Each item is scored on a 5-point Likert scale, with scores ranging from 0 (no issue) to 4 (extremely severe issue), leading to a total score that can vary from 0 to 28. In this study, the cutoff score was 7, indicating that a participant with a score of 7 or above had insomnia [32]. The Chinese version of ISI proves to be a reliable and valid instrument for adolescents [33]. The ISI showed a Cronbach's alpha of 0.71 in the current study.

#### 2.2.5. Aggression

Physical and verbal aggression was assessed using six selected items in the Short-Form Buss–Perry Aggression Questionnaire (BPAQ-SF). In the present study, wo items were selected for physical aggression (e.g., If people around me get me in trouble, I will probably get into a fight), and four items were selected for verbal aggression (e.g., I often feel at odds with others). Each item was assessed using a 5-point Likert scale, ranging from 1 (not applicable) to 5 (highly applicable) [34], with elevated scores reflecting a greater degree of aggression. This scale has shown strong reliability among Chinese adolescents [35]. The scale had a Cronbach's alpha of 0.85 in this study.

### 2.3. Statistical Analysis

In this research, LPA was conducted using Mplus 8.6 (Muthén & Muthén, Los Angeles, CA, USA) by examining the scores of both physical and verbal aggression. To determine the most suitable model, various criteria were employed, including the Akaike information criterion (AIC), Bayesian information criterion (BIC), sample size adjusted BIC (aBIC), Lo–Mendell–Rubin (LMR) likelihood ratio test, bootstrapped likelihood ratio test (BLRT), and entropy metrics. During the modeling process, a starting model (with only one class) was established first, and it evolved with an increasing number of classes included until the optimal fitting model was established. There were three main evaluation criteria for the latent profile model fitting index: (1) the model with the lowest AIC, BIC, and aBIC was regarded as the best model; (2) an entropy closer to 1 indicated higher accuracy of classification; and (3) the likelihood based on BLRT and LMR was used to compare the model with *n* categories and that with *n*−1 categories, with significant values indicating that the model with *n* categories was better than that with *n*−1 categories.

The *χ*^2^ test served to compare categorical variables between groups. Continuous data were represented by means and standard deviations (SDs), while nonparametric methods were utilized for comparisons between groups. To evaluate the relationship between aggression and insomnia, logistic regression analysis was conducted. With regard to modeling, Model 1 was used to calculate the unadjusted odds ratio (OR); Model 2 was used to obtain the OR with adjustment for covariates such as age, sex, and ethnicity; and Model 3 was used to obtain OR with adjustment for anxiety and depressive states in addition to age, sex, and ethnicity. To examine the pattern of relationship between the aggression score and the risk of insomnia after adjustment for age, sex, ethnicity, anxiety, and depression, a restricted cubic spline model was applied. The statistical analyses were conducted with the use of SPSS software (version 26.0; IBM Corp., Chicago, IL), establishing a significance level of *p*  < 0.05 (two-tailed). The analyses based on the restricted cubic spline model were conducted using R software (version 4.2.3).

## 3. Results

**Figure 1:**
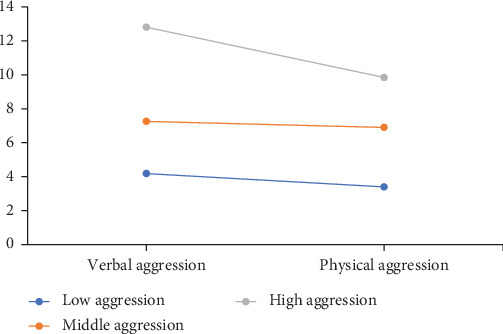
Scores of different levels of aggression based on latent profile analysis.

According to the LPA (Table 1), as the model's profiles rise from 1 to 4, AIC, BIC, and aBIC values consistently decline, while the entropy shows a continuous increase. The LMR for the four-class model was found to be insignificant (*p*  > 0.05) and therefore excluded. Thus, the model comprising three classes was ultimately identified as the most suitable model for this study. The average latent probabilities for profiles 1, 2, and 3 within the three-class model were recorded at 97.6%, 100%, and 99.3%, respectively, indicating strong discriminative ability and reliability of the LPA for the three-class model. As illustrated in Figure 1, class 1 (*n* = 931, 82.8%) exhibited the lowest levels of aggression and was classified as the low-aggression profile, class 2 (*n* = 153, 13.6%) was characterized by medium scores of physical and verbal aggression and was regarded as the moderate-aggression profile, and class 3 (*n* = 40, 3.6%) displayed the highest scores of aggression and was regarded as the high-aggression profile.

Table 2 illustrates the comparison of demographic and clinical traits among the low-, moderate-, and high-aggression profiles. Notable differences were observed in the proportion of participants experiencing baseline depression, baseline anxiety, and insomnia in 8 months between the three profiles (all *p* < 0.05). High aggression profile has the highest percentage of depression (47.5%), anxiety (30.0%), and insomnia (65.0%) individuals compared to the other profiles. Nonetheless, there were no notable differences observed among the three groups regarding sex, age, and ethnicity.

**Figure 2:**
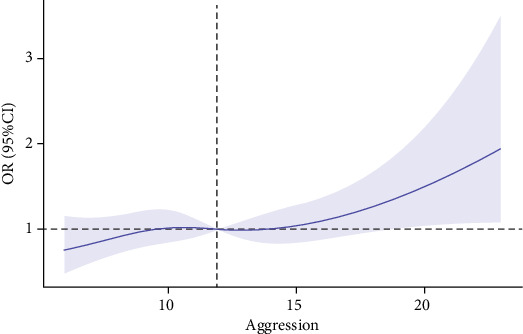
Cubic model of the association between aggression scores and the risk of insomnia after adjustment for age, sex, ethnicity, anxiety, and depression.

The follow-up survey after 8 months found 228 (20.3%) new cases of insomnia. Moreover, the associations between different levels of aggression and insomnia are presented in Table 3. Compared with participants exposed to a low level of aggression, the crude OR of insomnia in adolescents with a moderate level of aggression was 6.77 (95%CI: 4.68–9.79), and the crude OR in adolescents with a high level of aggression was 12.09 (95%CI: 6.14–23.78) (Model 1). After adjustment for covariates (age, sex, and ethnicity), the OR of insomnia in participants with a moderate level of aggression was 6.80 (95%CI: 4.68–9.89), and the OR in participants with a high level of aggression was 12.51 (95%CI: 6.32–24.76), as compared with those with a low level of aggression (Model 2). After adjustment for age, sex, ethnicity, anxiety, and depression, the associations of moderate and high levels of aggression with insomnia were still significant, with ORs of 6.10 (95%CI: 4.17–8.93) and 9.98 (95%CI: 4.94–20.15), respectively. In the model of restricted cubic spline regression, the pattern of the association between aggression and the risk of insomnia was linear (Figure 2). In this study, the risk of insomnia significantly increased when the aggression score was greater than 12.

## 4. Discussion

This prospective cohort study showed an association between different severity levels of aggression and insomnia in Chinese adolescents. Specifically, our findings indicated that moderate and high levels of aggression were linked to a significantly elevated risk of insomnia, even after controlling for age, sex, ethnicity, anxiety, and depression. In addition, the restricted cubic spline model indicated a linear relationship between aggression and the risk of insomnia.

According to the results of LPA of aggression in behavior dimension, 82.8% (*n* = 931) of the adolescents were classified into the low-aggression profile, while 13.6% (*n* = 153) and 3.6% (*n* = 40) were categorized as moderate-aggression and high-aggression profiles, respectively. The present study identified three distinct profiles, all of which are both theoretically meaningful and statistically valid. These findings are consistent with prior research. For example, a previous study on children and adolescents' aggression identified three distinct profiles of reactive and proactive aggression: “severe aggressive” (4.23%), “highly aggressive” (15.41%), and “moderately low aggressive” (80.36%) [36]. These proportions are closely aligned with the profile distributions observed in the present study. Additionally, we found that 17.2% of the participants in the current study exhibited moderate or high levels of aggression. This result is comparable to findings from a large-scale community survey in the United States, which reported that 17% of adolescents and 8% of adults experienced recurrent aggressive behaviors [37]. Furthermore, previous studies have shown that aggressive behaviors are relatively common among Chinese adolescents, with prevalence rates ranging from 15.7% to 24.3% [14, 15]. However, differences in sample characteristics and measurement methods between studies should be considered to avoid overgeneralizing the findings. Despite these methodological variations, the consistency in aggression profile distributions is noteworthy. Together, these findings confirm that aggression is prevalent during adolescence. During this period, individuals undergo significant changes in brain development, particularly in the fronto-limbic-striatal circuit, which is related to impulse control and decision-making [9].These changes may increase their susceptibility to aggressive behaviors. Social pressures, identity formation challenges, and heightened sensitivity to peer influence further contribute to increased conflict and aggression in this period [38–40]. Thus, identifying these distinct aggression profiles may provide a framework for targeted interventions, enabling early detection and treatment of adolescents at risk of adverse outcomes such as insomnia.

The present study also found that participants exhibited moderate and high levels of aggression were more prone to experiencing depression and anxiety, consistent with prior research showing that aggression frequently co-occurs with emotional disorders, such as depression and anxiety [41, 42]. Longitudinal studies have further demonstrated a bidirectional relationship between aggression and emotional disorders, suggesting that each can reinforce the other over time [43]. Several theoretical models can explain these associations. The “acting-out model” suggests that internalized problems, such as anxiety and depression, may eventually manifest as externalized behaviors, including aggression [44, 45]. Similarly, the “failure model” posits that adolescents who engage in aggressive behavior often encounter increased psychosocial challenges, such as rejection from peers and parents, which in turn heightens their vulnerability to developing depression and anxiety [46]. Finally, the “reciprocal model” highlights the bidirectional nature of the relationship between depression and aggression, emphasizing that shared factors, such as maladaptive coping styles, can reinforce both conditions over time [47].

The present study also found that moderate and high levels of aggression were significantly associated with an increased likelihood of experiencing insomnia. Prior studies have shown that sleep disturbances, including insomnia, can contribute to aggression through emotional dysregulation, cognitive impairments, and inhibitory control deficits [48]. For example, one study found that shorter sleep duration is associated with increased physical aggression among urban adolescents [49]. Similarly, verbal aggression has also been linked to sleep problems, including insomnia [50]. However, fewer studies have explored the reverse relationship—namely, how aggressive behaviors may impair sleep—particularly in adolescents. For example, a study on juvenile offenders reported that aggression was a significant predictor of sleep problems [51]. Similarly, other research has suggested that aggression disrupts sleep quality by increasing stress and physiological arousal [20, 52]. Consistent with this evidence, our findings revealed that adolescents with higher levels of aggression had an elevated risk of insomnia. The arousal theory offers a plausible mechanism to explain this association. It posits that aggressive behaviors trigger physiological arousal, which activates the sympathetic nervous system as part of a fight-or-flight response [53]. This response is regulated by the hypothalamic–pituitary–adrenal (HPA) axis [53]. Since persistent sympathetic activation interferes with the ability to relax and transition into sleep, adolescents with heightened aggression may struggle to fall asleep and maintain sleep throughout the night [54]. In addition to physiological arousal, social stressors play a critical role in the relationship between aggression and insomnia. Adolescents are often involved in peer interactions that may involve conflict, victimization, or bullying [55]. These experiences can create anxiety about personal safety and generate a sense of threat, both of which impair sleep quality [56]. Furthermore, these negative social interactions can lead to hypervigilance—a heightened state of awareness—making it even more difficult for adolescents to relax and initiate sleep [57]. Collectively, these findings highlight the need for educational and clinical interventions aimed at addressing aggression-related sleep problems among adolescents to improve their overall mental health and well-being.

This research presents a few limitations. Firstly, the sample was drawn from a specific region in China, potentially restricting the ability to generalize the results to different populations. Additionally, the dependence on self-reported measures for aggression and insomnia might have introduced reporting bias, which affects the accuracy of results. Lastly, other variables related to insomnia, such as a history of smoking or drinking, might need to be included.

## 5. Conclusion

The present study showed that the LPA effectively classified participants into three profiles based on physical and verbal aggression. Additionally, the findings indicated that higher levels of aggression were associated with an increased risk of insomnia. These findings highlight the importance of early identification and intervention for adolescents exhibiting aggressive behaviors, as timely intervention may help prevent the development of insomnia. Schools and mental health professionals could implement integrated interventions that focus on aggression management and emotional regulation to improve both behavioral and sleep outcomes. Cognitive behavioral therapy targeting aggression, in combination with mindfulness practices or relaxation techniques, could be particularly effective in reducing hyperarousal and improving sleep outcomes. Furthermore, peer mediation programs may help adolescents develop healthy interpersonal skills and reduce the social stressors that can contribute to insomnia.

## Figures and Tables

**Table 1:** Model fit indices for determining the optimal number of classes.

Model	AIC	BIC	aBIC	Entropy	LMR	BLRT	Class proportion
1	9463.453	9483.552	9470.847	—	—	—	—
2	8081.012	8116.185	8093.951	0.956	<0.001	<0.001	0.864/0.136
3	7182.655	7232.902	7201.139	0.979	0.018	<0.001	0.136/0.036/0.828
4	6881.253	6946.573	6905.282	0.983	0.068	<0.001	0.146/0.005/0.814/0.035

*Note:* The values in the LMR and BLRT columns are the *p* values related to LMR and BLRT in comparison of model fit.

Abbreviations: aBIC, adjusted Bayesian information criterion; AIC, Akaike information criterion; BIC, Bayesian information criterion; BLRT, bootstrapped likelihood ratio test; LMR, Lo–Mendell–Rubin.

**Table 2:** Demographic and clinical characteristics of participants with low, moderate, and high levels of aggression.

		Low *N* = 931	Moderate *N* = 153	High *N* = 40	F/*χ*^2^	*p*
Age mean (SD)	—	18.14 (0.76)	18.03 (0.70)	18.00 (0.72)	1.96	0.141
Sex (%)	Male	563 (60.5)	84 (54.9)	29 (72.5)	4.343	0.115
	Female	368 (39.5)	69 (45.1)	11 (27.5)	—	—

Ethnicity (%)	Han	826 (88.7)	129 (84.3)	38 (95)	4.264	0.119
	Minority	105 (11.3)	24 (15.7)	2 (5)	—	—

Depression (%)	No	908 (97.5)	127 (83.0)	19 (47.5)	51.92	<0.001
	Yes	23 (2.5)	26 (17.0)	21 (52.5)	—	—

Anxiety (%)	No	921 (98.9)	140 (91.5)	28 (70.0)	38.216	<0.001
	Yes	10 (1.1)	13 (8.5)	12 (30.0)	—	—

Insomnia in 8 months (%)	No	807 (86.7)	75 (49.0)	14 (35.0)	166.55	<0.001
	Yes	124 (13.3)	78 (51.0)	26 (65.0)	—	—

*Note:* Depression and anxiety were assessed in baseline.

**Table 3:** The associations between different levels of aggression and insomnia.

Aggression level	No	Pre (%)	Model 1			Model 2			Model 3		
			OR	95%CI	*p*	OR	95%CI	*p*	OR	95%CI	*p*
Low (ref)	931	82.8	1.00	—	—	1.00	—	—	1.00	—	—
Middle	153	13.6	6.77	4.68–9.79	<0.001	6.80	4.68–9.89	<0.001	6.10	4.17–8.93	<0.001
High	40	3.6	12.09	6.14–23.78	<0.001	12.51	6.32–24.76	<0.001	9.98	4.94–20.15	<0.001

*Note:* Model 1, crude OR; Model 2, OR adjusted for age, sex, and ethnicity; Model 3, OR adjusted for age, sex, ethnicity, anxiety state, and depressive state.

Abbreviation: Pre, prevalence.

## Data Availability

The data that support the findings of this study are available from the corresponding author upon reasonable request.

## Nomenclature

aBIC: Sample size adjusted BIC
AIC: Akaike information criterion
BIC: Bayesian information criterion
BLRT: Bootstrapped likelihood ratio test
BPAQ-SF: Short-Form Buss–Perry Aggression Questionnaire
GAD-7: 7-item Generalized Anxiety Disorder Scale
ISI: Insomnia Severity Index
LMR: Lo–Mendell–Rubin
LPA: Latent profile analysis
OR: Odds ratio
PHQ-9: Patient Health Questionnaire
SDs: Standard deviations.
